# Combined metabolomics and transcriptomics reveal the secondary metabolite networks in different growth stages of *Bletilla striata* (Thunb.) Reichb.f.

**DOI:** 10.1371/journal.pone.0307260

**Published:** 2024-07-24

**Authors:** Man Chen, Xia Wang, Yueyu Ye, Xiaomei Li, Shiqing Li, Meiya Li, Fusheng Jiang, Chunchun Zhang

**Affiliations:** 1 College of Pharmaceutical Sciences, Zhejiang Chinese Medical University, Hangzhou, China; 2 Academy of Chinese Medical Sciences, Zhejiang Chinese Medical University, Hangzhou, China; 3 College of Life Sciences, Zhejiang Chinese Medical University, Hangzhou, China; ICAR - National Research Center on Plant Biotechnology, INDIA

## Abstract

**Background:**

*Bletilla striata* (Thunb.) Reichb.f. (*B*. *striata*) is a traditional Chinese medicinal herb. *B*. *striata* polysaccharides (BSP), stilbenes and 2-isobutyl malic acid glucosoxy-benzyl ester compounds are the main active ingredients in *B*. *striata*. However, there is limited report on the changes of medicinal components and their biosynthesis regulation mechanisms in the tubers of *B*. *striata* at different stages.

**Method:**

The tubers of *B*. *striata* were collected during the flowering period, fruiting period, and harvest period to determine the total polysaccharide content using the phenol sulfuric acid method. The changes in secondary metabolites in the tubers at these stages were analyzed by ultra performance liquid chromatography tandem mass spectrometry (UPLC-MS), and transcriptomics was conducted for further exploration of their biosynthetic pathways.

**Result:**

The BSP content gradually increases from the flowering period to the fruiting period as the tubers develop, reaching its peak, but subsequently decreases at harvest time, which may be associated with the germination of *B*. *striata* buds in later stage. A total of 294 compounds were identified in this study. Among them, a majority of the compounds, such as 2-isobutyl malate gluconoxy-benzyl ester, exhibited high content during the fruit stage, while stilbenes like coelonin, 3’-O-methylbatatasin III, and blestriarene A accumulated during the harvesting period. The transcriptome data also revealed a substantial number of differentially expressed genes at various stages, providing a partial explanation for the complex changes in metabolites. We observed a correspondence between the expression pattern of GDP-Man biosynthesis-related enzyme genes and cumulative changes in BSP. And identified a positive correlation between 9 transcription factors and genes associated with polysaccharide biosynthesis, while 5 transcription factors were positively correlated with accumulation of 2-isobutyl malate gluconoxy-benzyl ester compounds and 5 transcription factors exhibited negative correlated with stilbene accumulation.

**Conclusion:**

It is imperative to determine the appropriate harvesting period based on the specific requirements of different active ingredients and the accumulation patterns of their metabolites. Considering the involvement of multiple transcription factors in the biosynthesis and accumulation of its active ingredients, a comprehensive investigation into the specific regulatory mechanisms that facilitate high-quality cultivation of *B*. *striata* is imperative.

## Introduction

*Bletilla striata* (Thunb.) Reichb.f. is a perennial herbaceous medicinal plant belonging to the Orchidaceae family. It primarily thrives in regions of southern and eastern China adjacent to the Yangtze River, as well as Japan, Korea, Vietnam, Thailand, and Myanmar [1, 2]. Its desiccated tubers have been utilized as a traditional Chinese medicine (TCM) for millennia, with its first mention recorded in Shennong’s Herbal Classic. It has the effect of astringent, hemostasis, detumescence and promoting granulation, and has high medicinal value in treating hemoptysis, hematemesis, trauma bleeding, sore swollen poison, and chapped skin [3].

The plant secondary metabolites are low molecular weight organic compounds that are not essential for the growth and development of plants [4]. Their formation is the result of long-term coevolution, evolution, and continuous adaptation between plants and their environment. Furthermore, these secondary metabolites exhibit diverse biological functions and play a pivotal role in regulating plant physiology, enhancing plant adaptability to physical environments, fortifying resistance against natural enemies, as well as bolstering defense mechanisms against diseases [5]. For instance, researchers have discovered a unique secondary metabolite called stilbene in orchids–a natural phytotoxin–which enhances plants’ ability to withstand external stressors [6].

Secondary metabolites in medicinal plants play a crucial role as the material basis for clinical efficacy or as key indicators for evaluating the quality of medicinal materials [7, 8]. Currently, it has been reported that hundreds of compounds including stilbenes (bibenzyl and phenanthrene), flavonoids, triterpenoids, steroids, monophenols and 2-isobutyl malic acid glucosoxy-benzyl ester compounds have been isolated from *B*. *striata*, which play pharmacological effects [1, 9]. The primary functional component and extensively investigated bioactive compound in the tuber of *B*. *striata* is the *B*. *striata* polysaccharide (BSP), which is predominantly composed of mannose and glucose. BSP demonstrates a wide range of biological activities, including anti-inflammatory and antioxidant effects, promotion of wound healing, facilitation of coagulation, and enhancement of immune response [10, 11]. Therefore, it has extensive applications in clinical treatments. For example, the *B*. *striata* polysaccharide hydrogel has demonstrated its effectiveness in promoting wound healing and can be used as an efficient wound dressing [12]. In addition to polysaccharides, tubers also contain compounds abundant in 2-isobutyl malate gluconoxy-benzyl ester and stilbene. The stilbene compounds present in *B*. *striata* exhibit antibacterial, anti-inflammatory, and antitumor properties. The phenanthrenes isolated from *B*. *striata* root ethanol extract showed bioactivity against *Staphylococcus aureus* [13]. Twenty-two bibenzyl compounds isolated from the ethyl acetate extract of *B*. *striata* showed moderate to good antibacterial activity against gram-positive strains [9], these literatures showed that phenanthrene and bibenzyl compounds may be the main pharmacological substances for the antibacterial effect of *B*. *striata* [14]. Pharmacological investigations have demonstrated the efficacy of extracts containing stilbene components from *B*. *striata* in preventing pulmonary fibrosis, while the antibacterial activity of stilbene against *Cutibacterium acnes* can be utilized for acne treatment [15]. The compound militarine belongs to the class of 2-isobutyl malate gluconoxy-benzyl ester, which is derived from 2-isobutyl malate, which is believed to have the effects of improving cognitive function in rats with chronic cerebral ischemia [16] and relaxing the isolated thoracic aorta rings of rats [17]. Currently, a total of 24 2-isobutyl malate gluconoxy-benzyl ester compounds have been isolated and identified in plants belonging to the Orchidaceae family [18].

In recent years, research on *B*. *striata* has primarily focused on its chemical composition, pharmacological actions, germplasm resources, cultivation and breeding of seedlings, clinical applications, etc. However, there has been limited investigation into its genetic composition. To address this gap in knowledge, some scholars have conducted omics studies on *B*. *striata*: Xu et al. [19] utilized Illumina double-terminal sequencing technology to construct a comprehensive nucleotide database for *B*. *striata*. By analyzing different transcriptome data of *B*. *striata*, researches have uncovered some molecular mechanisms underlying organ development, stress resistance, and accumulation of medicinal components. Previous studies partially addressed the lack of genetic information annotation in the non-model species *B*. *striata* and explored metabolic pathways for some of its secondary metabolites. However, these studies primarily relied on uniomics analyses and did not effectively reveal the genetic information of *B*. *Striata*.

In the current era of rapid advancements in high-throughput sequencing, the integrating metabolome and transcriptome analysis has emerged as a prevalent approach for investigating secondary metabolite composition, identifying gene function, and elucidating metabolic pathways during plant development [20]. For example, by integrating metabolomics and transcriptome data, key genes involved in the biosynthesis of water-soluble polysaccharides (WSP), flavonoids, and phosphoserine in leaves of *Dendrobium officinale* at different developmental stages were identified by integrating metabolomics and transcriptome data [21]. Additionally, gene regulation and metabolic processes during floral development in *Dendrobium officinale* have been explored to elucidate the molecular mechanisms underlying floral development [22].

By integrating transcriptomic sequencing and non-targeted metabolic profiling data from different organs of *B*. *striata*, five different metabolic pathways for polysaccharide, sterols, flavonoids, terpenes, and alkaloids biosynthesis in *B*. *striata* have been tentative explored in previous studies [23]. However, the regulatory changes of BSP and secondary metabolites during the development of *B*. *striata* tubers are remain unclear. Therefore, this study aims to investigate the cumulative changes in BSP and secondary metabolites throughout different developmental stages of *B*. *striata* tubers, as well as to explore the characteristics of their biosynthetic regulatory network.

## Methods and material

### Plant materials

My study did not involve human participants, specimens or tissue samples, or vertebrate animals, embryos or tissues:

1. We state clearly that no specific permissions were required for these locations/activities, and provide details on why this is the case. The samples were collected from Anji County laboratory planting base in Zhejiang Province, where we have carried out related research on the cultivation of *Bletilla striata* (Thunb.) Reichb.f.
2. We confirm that the field studies did not involve endangered or protected species.
3. We confirm that the anthors had received approval from Anji County laboratory planting base in Zhejiang Province to collect samples from the plants.

The contents of secondary metabolites and polysaccharides in three developmental stages of *B*. *striata* tubers were studied: HQ (flowering period, late April), GQ (fruiting period, late September), and CSQ (harvest period, late October). Plant samples were collected from Anji County laboratory planting base, Zhejiang Province, and authenticated by Associate Professor Shui-li Zhang from the College of Pharmaceutical Sciences at Zhejiang Chinese Medical University. Subsequently, the samples were frozen at -80 °C in a refrigerator for further use. A voucher specimen (BS20210909) was stored in the laboratory of the College of Pharmaceutical Sciences, Zhejiang Chinese Medical University.

### Determination of polysaccharide content

20 g of *B*. *striata* sample was weighed (5 tubers derived from 5 different plants were combined as one biological sample) and 200 mL of water was added to homogenization for 2 min, filtered using an 80 mesh screen, and the mixture was freeze-dried subsequently. 200 mg of dried sample was weighed and placed in a 15 mL centrifuge tube, and 5 mL of 50% ethanol was added. Thoroughly mixed, standing for 30 min, then ultrasonic extracted (53 kHZ, 250 W) for 15 min, centrifuge at 8,000 rpm at 4 °C for 5 min. The supernatant was collected for metabolomics analysis, while the precipitate was resuspended in 20 mL of water and subjected to ultrasound extraction (53 kHZ, 250 W) in a water bath at 90) for 2 h. An equal volume of anhydrous ethanol was added to precipitate the polysaccharides, followed by centrifugation at 8000 rpm at 4 °C for 5 min. The resulting precipitate was dissolved in 5 mL of water as the test sample, with three replicates per group. Anhydrous D-glucose (25.0 mg) was used to prepare a glucose standard stock solution with a mass concentration of 1 mg/mL by adjusting the volume with ultra-pure water to reach a final volume of 25 mL. Subsequently, a series of glucose solutions were prepared with concentrations ranging from 0.05 to 0.4 mg/mL (0.05, 0.1, 0.2, 0.3, 0.4 mg/mL). The content was determined at 490 nm using the phenol-sulfuric acid method. A standard curve was generated by plotting glucose concentrations on the Y-axis against absorbance values on the X-axis, followed by quantification of polysaccharide content [24].

### Metabolome analysis

The supernatant obtained during initial extraction process of polysaccharides, was transferred to a liquid phase vial for UPLC-MS detection and analysis. Three biological samples were included in each group. All samples were mixed equally to create quality control (QC) samples, while 50% ethanol was used as a blank sample.

The electrospray ionization (ESI) was used as the ionization source on a Q-TOF SYNAPT G2-Si High Definition Mass Spectrometer (Waters, UK). The separation was performed by a Waters ACQUITY UPLC Cortecs T3 Column (2.1× 50 mm, 1.7 μm) with Cortecs T3 Van Guard (2.1×50 mm, 1.7 μm). Briefly, in ESI-MS analysis, the capillary voltage was set as 3.0 kV. The MSE continuum mode was carried out over the range of m/z 50–1500. Each sample was injected 2 μL into the column and employed with a gradient elution. The column and autosampler temperature were maintained at 30 °C and 25°C, respectively. The gradient elution consisted of A (acetonitrile) and B (0.1% formic acid in water), the flow rate was controlled at 0.35 mL/min. The gradient program was set as follows: 5%A→20%A (0–4 min), 20%A→25%A (4–7 min), 25%A (7–8.5 min), 25%A→42%A (8.5–17 min), 42%A→85%A (17–27 min), 85%A→ 100%A (27–28 min), 100%A (28–30 min).

The data files were identified, extracted and aligned by Prodenesis QI software. Characteristic peaks with a coefficient of variation (CV) greater than 30% in quality control (QC) samples were excluded. The remaining characteristic peaks were compared against both the self-built *B*. *striata* database and online public databases to identify the compounds. The compound classification was performed using https://npclassifier.ucsd.edu/. Multivariate statistical analysis of metabolites was conducted through Maiwei Cloud platform. Differentially accumulated metabolites (DAMs) were identified based on a Fold Change >1.5 and a variable importance in projection (VIP) >0.8, followed by visualization using R and Origin.

### RNA extraction and transcriptome analysis

Total RNA was extracted using RNAprep Pure Plant Plus Kit (Tiangen Biotech, Co., Beijing, China), total RNA content and quantity were detected by Agilent 2100 analyzer (Agilent Technologies Co Ltd., USA.), cDNA library was prepared by NEBNext Ultra RNA library kit (Shenzhen Micro Science and Technology Group Co., Ltd., Shenzhen, China), and effective concentration of library was accurately quantified by qRT-PCR. High-throughput sequencing using Illumina Novaseq6000, and three biological replicates per group were applied. Low-quality reads were filtered out by Trimmomatic (0.39), and transcripts of clean reads were spliced by Trinity (v2.1.1) to serve as reference sequences for subsequent analysis [25, 26]. After selecting Corset (Version1.09) hierarchical clustering, the longest cluster sequence is obtained as Unigene and BUSCO (v5.1.2) for concatenation quality evaluation. Nr, Nt, KOG, Swiss-prot, Uniprot, KEGG and GO seven databases were selected for functional annotation of Unigene [27, 28]. The RNA clean reads were aligned to the reference genome using RSEM (vl2.28) [29]. The TPM value was utilized for the quantification of gene expression levels, while DEseq2 was employed to identify differentially expressed genes. The differential genes were selected based on a significance threshold of *p* < 0.05 and |log2 (Fold Change) | ≥ 1.

### Combined transcriptome and metabolome analysis

To gain a thorough understanding of the regulatory network underlying secondary metabolite biosynthesis in *B*. *striata*, we performed an integrated analysis of both metabolome and transcriptome data. This analysis aimed at exploring the relationship between differentially detected metabolites and genes involved in their respective biosynthetic pathways, as well as identifying transcription factors that govern secondary metabolism. As a result, we obtained a correlation data matrix which was then used to construct a comprehensive correlation network map.

### Identification of transcription factors (TFs)

iTAK software is used to predict plant transcription factors. The basic principle is to identify TFs with hmmscan by using TF-transcription factor family and rules defined by classification in database.

### Statistical analysis

The statistical analysis was performed using GraphPad Prism 8.0 (GraphPad Software, La Jolla, CA, USA) for data processing, statistical analysis, and plotting. One-way ANOVA followed by Tukey’s test was used for group comparisons. An independent-samples t-test was employed to analyze differences between the two groups. Statistical significance was defined as *p* < 0.05.

## Results

### Changes of polysaccharide content and buds of *B*. *striata* in different developmental periods

**Fig 1:**
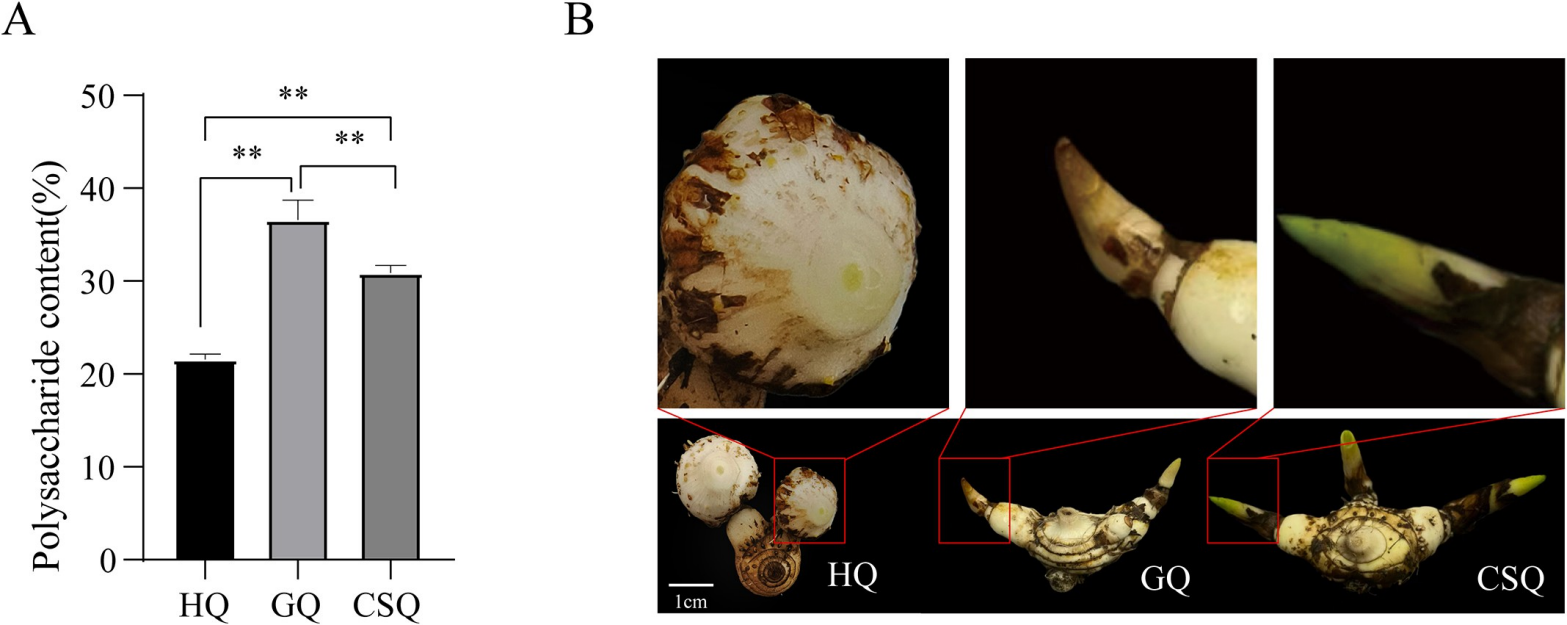
Changes in BSP content during different developmental stages of *B*. *striata* tubers (n = 3), **, *P* ≤ 0.01 (A); The morphology of tubers and buds during the flowering (HQ), fruiting (GQ) and harvesting periods (CSQ) of *B*. *striata* (B).

Utilizing glucose as the reference standard, a linear regression equation was calculated as *Y* = 3.9767*X* + 0.2131 (R^2^ = 0.9995). The polysaccharide content of *B*. *striata* at different developmental stages was analyzed. As depicted in Fig 1A, the polysaccharide content of *B*. *striata* tubers during the flowering stage, fruiting stage and harvest stage were determined to be 22.25%, 36.62% and 31.79% respectively. Obviously, there was a significant increase in polysaccharide content from the flowering to fruiting stage; however, it slightly decreased during the harvest period while still remaining significantly higher than that observed during flowering stage levels. By comparing the morphology of tubers and buds between fruiting and harvest periods (Fig 1B), it is apparent that the new buds of the latter were significantly larger than those of the former.

### Multivariate statistical analysis and differential metabolite screening

**Fig 2:**
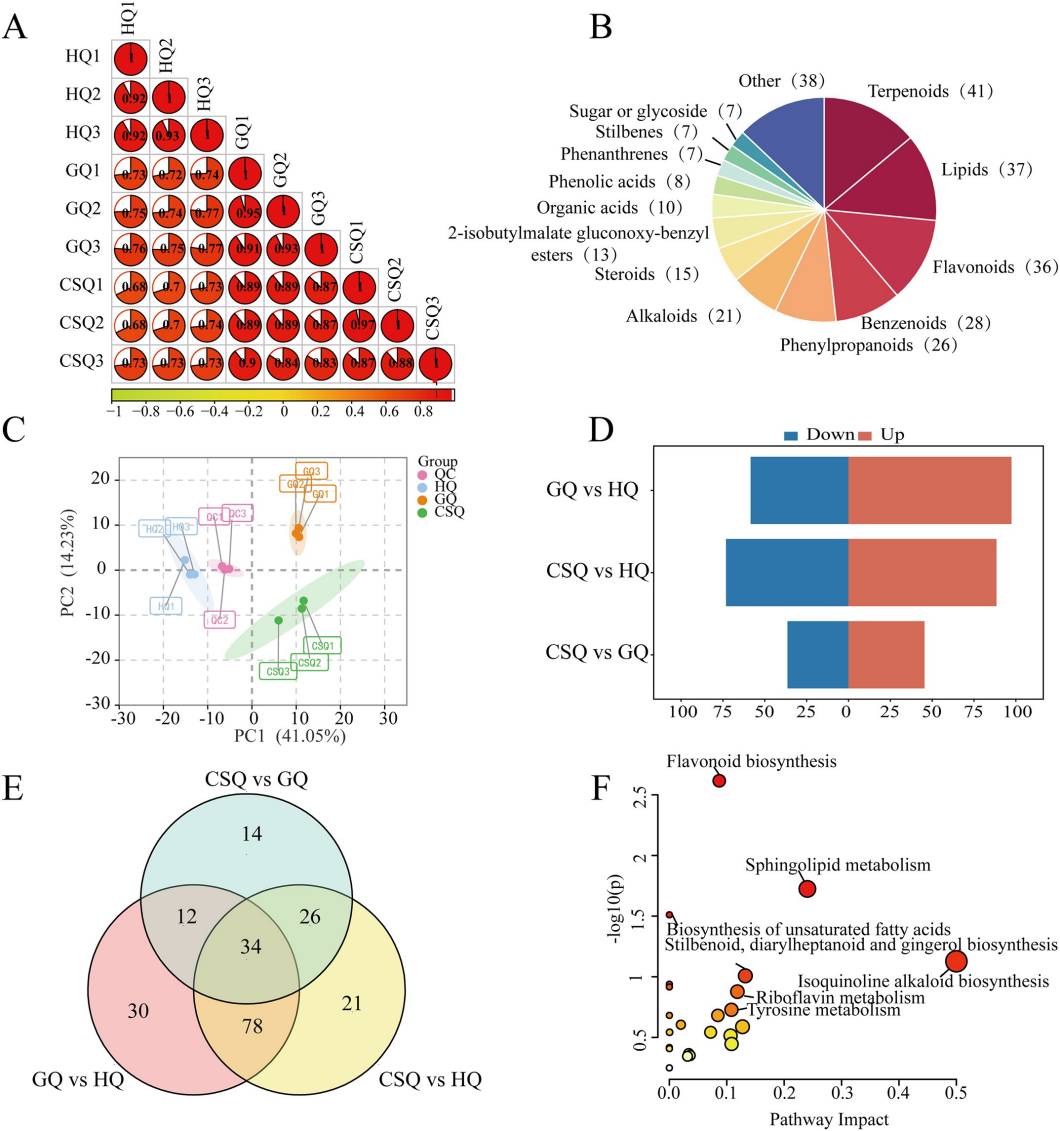
Analysis of metabolites of *B*. *striata* tuber in different developmental periods. Intra-group and inter-group correlations of 9 samples at different developmental stages (A); Classification and composition of secondary metabolites (B); PCA Analysis (PC1 = 41.05%, PC2 = 14.23%) (C); Histogram of the number of differential metabolites between three different comparison groups (D); Venn diagram of differential metabolites between different comparison groups (E); KEGG pathway analysis of all differential metabolites (F).

In order to investigate the dynamics of secondary metabolites during the growth of *B*. *striata*, a non-targeted metabolomics analysis was conducted on tuber samples collected at three distinct growth and developmental stages: flowering, fruiting, and harvest. The UPLC-MS/MS system was employed for detecting metabolites in both positive and negative ion modes. Additionally, equal volumes of these samples were mixed to prepare quality control (QC) samples for assessing method stability. S1 Fig presents the total ion chromatograms (TIC) obtained from QC samples in positive and negative ion modes. The high correlation coefficient indicates excellent repeatability and reliability of the data, enabling further analysis (Fig 2A).

A total of 294 metabolites were identified (S1 Table), including terpenes, alkaloids, 2-isobutyl malate gluconoxy-benzyl ester, stilbene compounds, etc. (Fig 2B). Unsupervised principal component analysis (PCA) was performed on the metabolomics data, which is an unsupervised pattern recognition method used to visualize the overall clustering trend of different groups and the degree of variation in the same group of samples. The results of PCA showed that all samples were clearly divided into three groups, and three biological replicates in each group were clustered together, confirming the reproducibility and reliability of the metabolomics method. PC1 and PC2 explained 41.05% and 14.23% of metabolite changes, respectively (Fig 2C), with a cumulative interpretation rate of 55.28%. The QC samples clustered around the origin indicating instrument stability. Orthogonal Projections to Latent Structures Discriminant Analysis (OPLS-DA), a supervised multivariate statistical method establishing a relationship model between metabolites and groups, was employed to obtain more detailed differences between groups. The OPLS-DA model was established and subjected to 200 permutation tests (S2 Table), which confirmed its reliability along with obtaining variable important in projection (VIP) values for all pairs of metabolites.

Using (VIP) > 0.8 and Fold Change >1.5 as screening criteria, we identified differential metabolites and generated volcano plots, which are presented in S2 Fig. In the three comparison groups, the number of differential metabolites was similar between GQ vs HQ and CSQ vs HQ, with 154 and 159 differential metabolites respectively. Among the GQ vs HQ comparison group, there were 95 up-regulated and 59 down-regulated differential metabolites. Similarly, in the CSQ vs HQ comparison group, there were 85 up-regulated and 74 down-regulated differential metabolites. The comparison group of CAQ vs GQ had relatively few differential metabolites, with 88 differential metabolites, of which 39 were up-regulated and 47 were down-regulated (Fig 2D). After plotting the overlapping differential metabolite data from each comparison group onto a Venn diagram, it was observed that there were 34 common types of differential metabolites across various comparison groups (Fig 2E). A total of 215 differential metabolites were identified from the three comparison groups. MetaboAnalyst 5.0 (https://www.metaboanalyst.ca/) was employed to conduct KEGG pathway analysis for all the identified differential metabolites (Fig 2F). The results showed that these differential metabolites were mainly enriched in "flavonoids biosynthesis", "sphingolipid metabolism", "unsaturated fatty acid biosynthesis", "isoquinoline alkaloid biosynthesis", etc. In addition, we noticed that some metabolites were also located in the "stilbenoid, diaryheptanoid and gingerol biosynthesis" pathway.

### Changes in metabolites of *B*. *striata* tuber at different developmental periods

**Fig 3:**
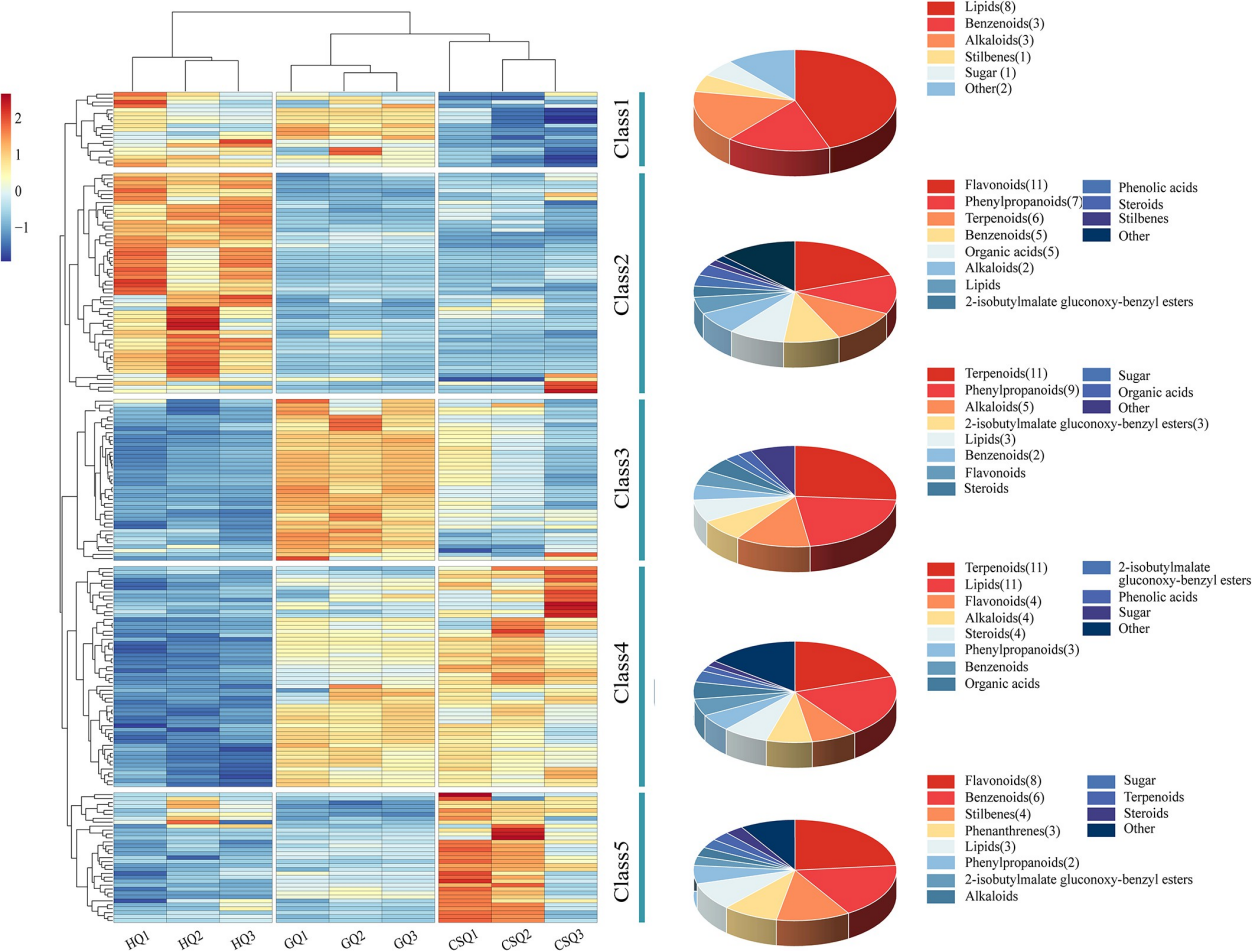
Heat maps of different metabolites of *B*. *striata* tuber at different developmental periods.

We visualized the trend of all the different metabolites using cluster heat maps (Fig 3), and found that the trend of differential metabolites can be divided into five main classes. The first class of metabolites, consisting of 18 compounds, exhibited a decline in levels during the harvest period. This group included 8 lipids, 3 benzenes, and 3 alkaloids, as well as 4 other categories such as mannose and batatasin III. The second class of compounds showed higher abundance during flowering but decreased in content with growth and development. This category comprised 56 metabolites including 11 flavonoids, 7 phenylpropanoids, 6 terpenes, and 5 organic acids. The third class consisted a total of 42 compounds including11 terpenoids and 9 phenylpropanoids, in addition to three extra compounds (dactylorhin A, gymnoside X, and gymnoside IX) belonging to the group of 2-isobutyl malic acid glucosyloxy-benzyl esters. The levels of these compounds exhibited an initial increase followed by a subsequent decrease. Although their content decreased during the harvest period, it still exhibited an upward trend relative to the flowering stage. Therefore, the period from flowering to fruiting development may represent a critical phase for the accumulation of these compounds, reaching their peak during the fruiting stage. The levels of fourth class compounds were low during the flowering stage, but gradually increased during the fruiting and harvest stages. The number of lipid compounds and terpenoids was the highest, with 11 species each. The level of fifth class compounds exhibited a notable upward trend from fruiting to harvesting stages, encompassing 8 flavonoids, 6 benzene compounds, 4 stilbenes compounds, and as well as 3 phenanthrene compounds and so on. Despite an abundant presence of flavonoids and benzene compounds, their contents remained comparatively low whereas those for both stilbenes and phenanthrene compounds were notably higher. Stilbenes and phenanthrene compounds in *B*. *striata* serve as pivotal bioactive components underpinning its pharmacological activities. Henceforth, the marked rise in concentration levels for these fifth class compounds during harvest season is closely associated with the quality of *B*. *striata*; nevertheless, further investigation is warranted to elucidate its underlying accumulation mechanism. In addition, we identified a total of 19 different metabolites belonging to 2-isobutyl malic acid glucosoxy-benzyl esters and diethylene compounds, most of which exhibited lower levels during the flowering stage compared to the other two periods. For instance, dactylorhin A, militarine, gymnoside III, gymnoside X and gymnoside IX exhibited a consistent pattern with the variation in BSP content that initially increased and subsequently decreased. The levels of coelonin, 3’-O-methylbatatasin III, blestriarene A, gymnoside V, and other compounds exhibited an accumulation pattern concomitant with the growth and development of *B*. *striata*.

### Transcriptome data and gene annotation

**Fig 4:**
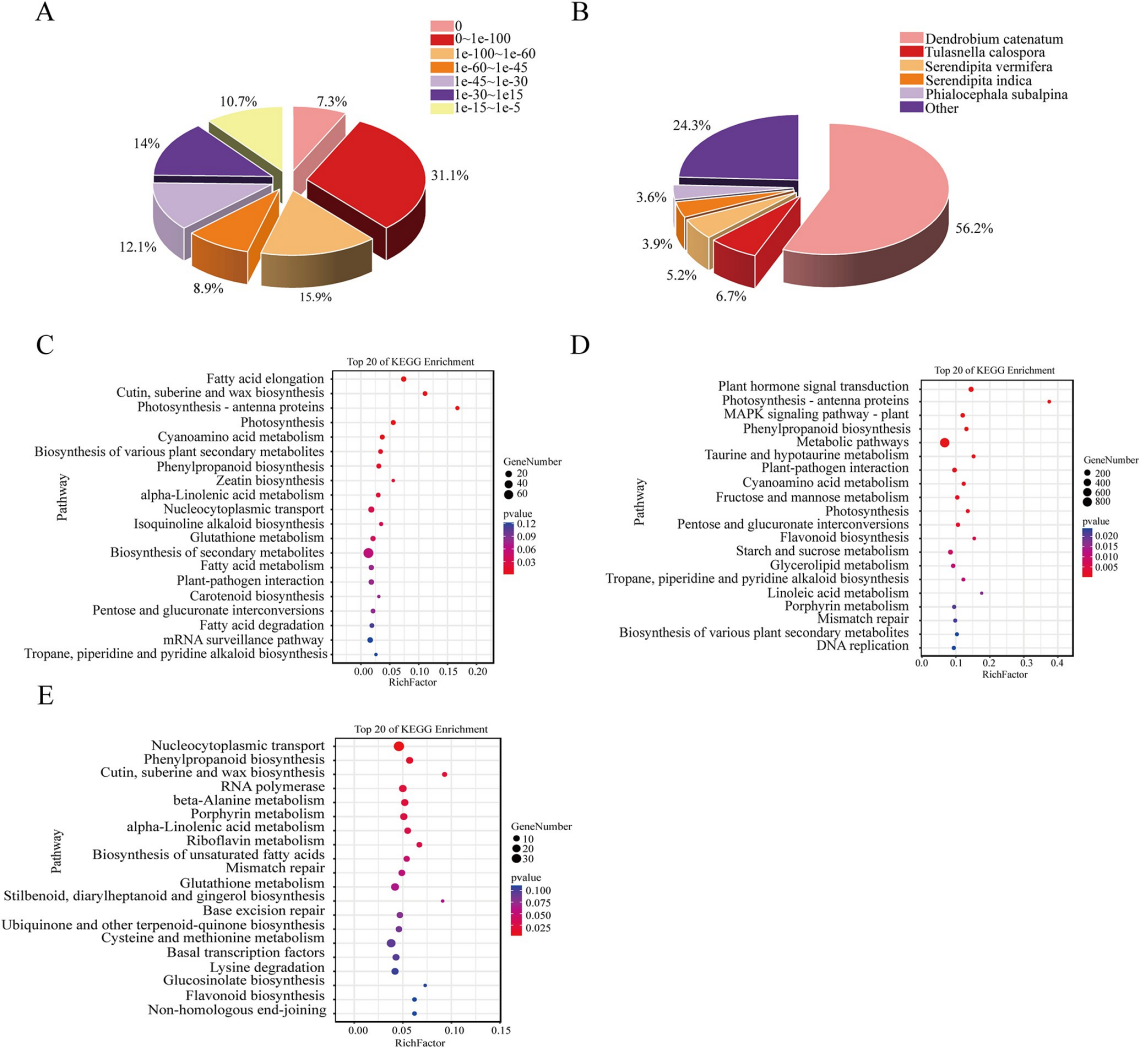
Transcriptome analysis of *B*. *striata* tuber at different growth and development stages. E-value distribution of unigenes in Nr database (A); Species similarity distribution of unigenes in Nr database (B); GQ vs HQ differential gene KEGG enrichment bubble map (C); CSQ vs HQ differential gene KEGG enrichment bubble map (D); CSQ vs GQ differential gene KEGG enrichment bubble map (E).

The transcriptome assay was conducted to investigate the transcriptome profiles of genes associated with secondary metabolite biosynthesis during tuber development in *B*. *striata*. In total, we obtained 52.14 GB clean data, consisting of 37,267,486–5,677,292 clean reads with a bases score (Q30) averaging between 99.89% and 99.91% (S3 Table). These reads were subsequently assembled into 237,252 transcripts and 176,598 unigenes. The length of unigenes in the range of 187–16537 bp, its N50 and N90 are 4755 bp and 2108 bp. the BUSCO evaluation showed a complete transcript of 93.7% (S3 Fig), indicating that the transcriptome assembly quality was good and subsequent analyses based on this data were reliable. These 176,598 unigenes were annotated through Nr, Nt, KOG, Swissprot, Uniprot, KEGG, and GO databases. NR, KOG, SwissProt and Uniprot annotated 118,020 (66.83%), 90,870 (51.46%), 83,337 (47.19%) and 83,244 (47.14%) sequentially similar genes, respectively. The annotation was conducted on 123,275 (69.81%) unigenes in at least one of the aforementioned seven databases. The comparison and annotation of the gene sequences with the Nr database provide significant insights into both the genetic similarity between this species and its related counterparts, as well as functional information associated with its genes. The Fig 4A depicts the distribution of E-values in the Nr database. The comparative analysis revealed that *B*. *striata* and *Dendrobium* exhibited the highest degree of gene annotation overlap in the Nr database, encompassing a total of 66,385 genes, which accounted for 56.2% of the annotated genes (Fig 4B).

The gene ontology classification revealed that 55,299 unigenes (31.31%) were categorized into three distinct groups: molecular function, cellular component, and biological processes. A detailed analysis of the biological processes group further classified them into “cellular process” (42,280 unigenes) and “regulation of gene expression” (37,192 unigenes). Within the cellular component group, the top three GO terms were “cytoplasm” (223,032 unigenes), “intracellular membrane-bounded organelle” (132,741 unigenes), and “nucleus” (112,524 unigenes). Further characterization of molecular function resulted in subclassification into “catalytic activity” (53,776 unigenes), “hydrolase activity” (37,947 unigenes), and “transferase activity” (37,604 unigenes). Using KOG analysis for functional categorization purposes yielded a grouping of 90,870 unigenes (51.46%) across 25 categories. The two most prominent categories were "general function prediction only" with 14,427 unigenes and "Posttranslational modification protein turnover chaperones" with 6,090unigens (S4 Fig).

### Differential gene screening and enrichment analysis

Based on |log 2 (Fold Change) | ≥1 and *p* value < 0.05, the number of GQ vs HQ, CSQ vs HQ and CSQ vs GQ differential genes was 1,278 (872 up-regulated, 406 down-regulated), 8,867 (6,152 up-regulated, 2,705 down-regulated) and 3,836 (2,174 up-regulated, 1,662 down-regulated), respectively. CSQ vs HQ had the largest amount of up-regulated and down-regulated differentially expressed genes (DEGs), and the difference was more significant. Compared with other groups, GQ vs HQ had the lowest amount of up-regulated and down-regulated DEG, followed by CSQ vs GQ. The findings revealed significant variations in gene expression levels during three distinct stages of tuber development in *B*. *striata*.

The top 20 enrichment pathways identified through KEGG enrichment analysis across the three comparison groups revealed significant enrichment of differential genes in flavonoid biosynthesis, unsaturated fatty acid biosynthesis, isoquinoline alkaloid biosynthesis, stilbenes, diarylheptane and ginger biosynthesis. These findings are consistent with the metabolomics results. In addition, differential genes of GQ vs HQ were significantly enriched in fatty acid elongation, cutin suberine and wax biosynthesis, photosynthesis and photosynthesis-antenna proteins (Fig 4C). The former two pathways are involved in the metabolism of fatty acids, which are essential lipids in plants. For example, long chain fatty acids are involved in cellular processes such as membrane transport, cell division and cell differentiation, and subsequently affect plant development [30]. While the conversion of fatty acids into cuticle and waxes serves to prevent water loss and provid protection against desiccation and external environmental stresses [31]. Notably, photosynthesis is a fundamental process that converts solar energy into chemical energy driving vegetative growth and development in plants [32]. In conclusion, the aforementioned pathways are intricately interconnected with plant development and aligning with the growth stages of *B*. *striata* from flowering to fruiting. CSQ vs HQ differential genes were significantly enriched in plant hormone signal transduction, photosynthesis-antenna proteins and MAPK signaling pathway-plant (Fig 4D). The growth and development of plants are regulated by hormonal cues, enabling the plant to systemically respond to environmental changes [33]. Meanwhile, the MAPK signal transduction pathway in plant individual growth and development and also play an important role in defense responses [34]. Evidently, these signaling pathways are associated with the growth and development of *B*. *striata* and may be implicated in mitigating environmental stressors such as high temperatures during July and August. CSQ vs GQ differential genes were significantly enriched in nucleocytoplasmic transport cysteine and phenylpropane biosynthesis, metabolism of some amino acids and biosynthesis of secondary metabolites like stilbenoid, diarylheptanoid and gingerol biosynthesis, flavonoid biosynthesis (Fig 4E). These pathways were consistent with the results of metabolome analysis during the fruiting and harvesting stages of tuber development in *B*. *striata*, which revealed an accumulation of secondary metabolites associated with the quality of *B*. *striata*. The KEGG enrichment results of the three different comparison groups revealed that the differentially expressed genes were enriched in the phenylpropane biosynthesis pathway, which is one of the primary secondary metabolic pathways in plants [35]. The pathway is intricately linked to plant growth and development. For instance, the production of flavonoids and stilbenes through this pathway can enhance plant resistance and actively participate in various fundamental processes during plant growth, including plant abiotic and biotic stress protection, plant–microbe interactions, or phytohormone transport and homeostasis [36].

### Analysis of transcription factors (TFs)

Transcription factors (TFs) are DNA-binding proteins that play an important role in plant growth and development by binding to specific DNA sequences called cis-elements in gene promoters [37, 38]. In this study, a total of 4,243 unigenes were annotated and assigned to 50 transcription factor families. Among these TF families, the MYB family exhibited the highest abundance with 432 unigenes, followed by C2H2 (363 unigenes), bHLH (257 unigenes), AP2/ERF (247 unigenes), C3H (247 unigenes), bZIP (226 unigenes) and WRKY (217 unigenes) (S5 Fig).

### Combined transcriptome and metabolome analysis

**Fig 5:**
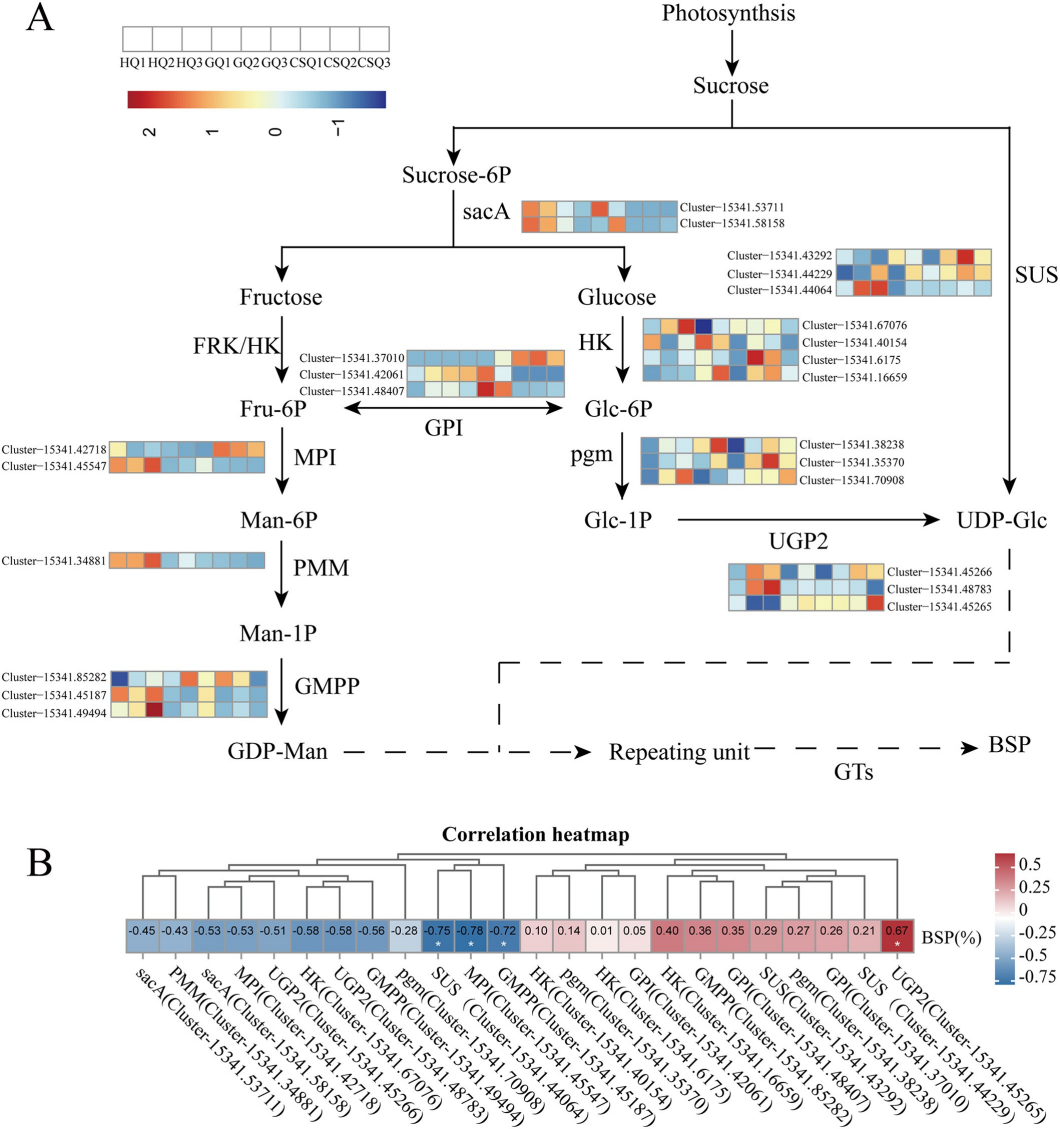
BSP biosynthetic pathway with related enzyme expression pattern and correlation. The temporal expression patterns of genes encoding enzymes involved in regulating BSP biosynthesis (A); Correlation between genes encoding enzymes involved in regulating BSP biosynthesis and BSP content (B). The first three colors are HQ samples, followed by GQ, and finally CSQ. Beta-fructofuranosidase (sacA), fyn-related kinase (FRK), hexokinase (HK), mannose-6-phosphate isomerase (MPI), phosphomannomutase (PMM), mannose-1-phosphate guanylyltransferase (GMPP), glucose-6-phosphate isomerase (GPI), phosphoglucomutase (pgm), UTP—glucose-1-phosphate uridylyltransferase (UGP2), sucrose synthase (SUS).

The abundance of stilbenes, 2-isobutyl malate gluconoxy-benzyl esters, and polysaccharide in *B*. *striata* contributes to their diverse pharmacological activities, establishing them as active ingredients of *B*. *striata*. Therefore, it is of great significance to analyze the changes of these metabolites and their corresponding metabolic genes. In order to identify the genes involved in the BSP biosynthesis pathway, based on the predominant monosaccharide components in BSP, we annotated the genes related to the metabolic pathway related to sugar. These pathways encompass starch and sucrose metabolism (ko00500), fructose and mannose metabolism (ko00051), as well as amino sugar and nucleotide sugar glycolysis metabolism(ko00520), glycolysis/gluconeogenesis (ko00010), etc. We have annotated a total of 24 genes that encode 9 enzymes, namely hexokinase (HK), mannose-6-phosphate isomerase (MPI), beta-fructofuranosidase (sacA), mannose-1- phosphate guanylyltransferase (GMPP), phosphomannomutase (PMM), glucose-6-phosphate isomerase (GPI), phosphoglucomutase (pgm), UTP-glucose-1-phosphate uridylyltransferase (UGP2), and sucrose synthase (SUS) associated with BSP biosynthesis (S3 Table). Among these genes, the expression of the two sacA-encoding genes was found to be higher during the flowering period compared to the other two stages. Additionally, a majority of the genes associated with GDP-Man production exhibited significantly elevated expression levels during the flowering stage. Conversely, most of the genes involved in either direct or indirect UDP-Glu production displayed heightened activity during both fruiting and harvest stages (Fig 5A). Correlation analysis between genes involved in BPS biosynthesis and polysaccharide content revealed the presence of nine enzymes that were significantly correlated with BSP content. Furthermore, the expression of UGP2 gene (Cluster-15341.45266) exhibited a positive correlation with BSP content, while the expression of SUS gene (Cluster-15341.44064), MPI gene (Cluster-15341.45547), and GMPP gene (Cluster-15341.45187) showed a negative correlation with BSP content (Fig 5B).

**Fig 6:**
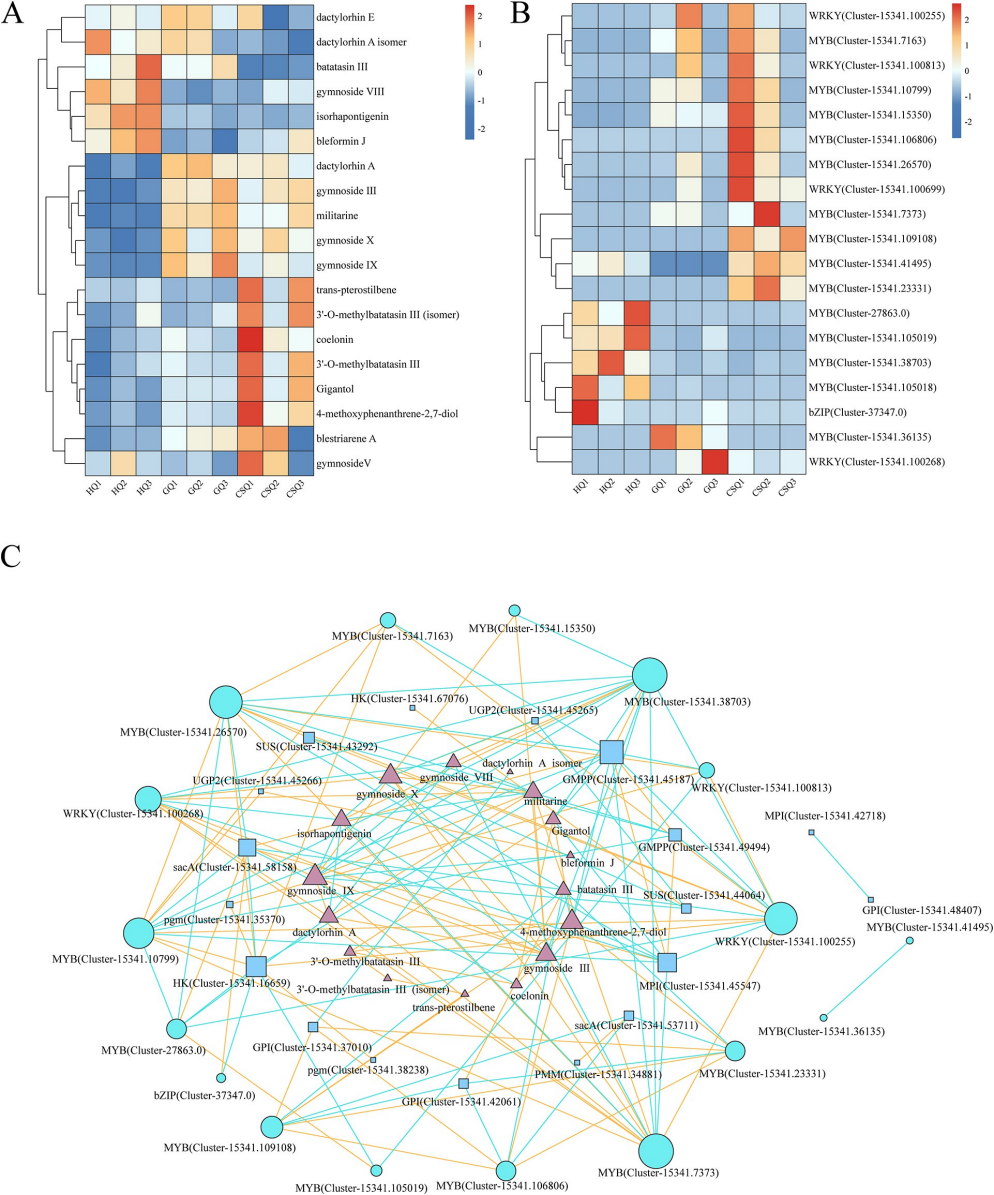
Correlation analysis of metabolite content with TFs expression and BSP biosynthesis genes. The changes of 2-isobutyl malate gluconoxy-benzyl esters and stilbenes in differential metabolites in different periods of tubers of *B*. *striata* (A); TFs expression in differential metabolites in different periods of tubers of *B*. *striata* (B); Correlation analysis network between metabolite content and transcription factor expression (C). The red line segment represents a positive correlation, while the blue line segment signifies a negative correlation).

The metabolome analysis revealed a higher abundance of secondary metabolites during the fruiting and harvesting stages, as depicted in Fig 6A. It is worth noting that there was a significant increase in fifth class compounds (Fig 3), especially stilbenes and phenanthrene compounds, during the harvesting period, which may be linked to the formation of *B*. *striata* quality. KEGG enrichment analysis of differentially expressed genes in the transcriptome showed a significant enrichment of the phenylpropanoid biosynthetic pathway, which is involved in the biosynthesis of flavonoids, stilbenes, and phenanthrene. However, most of these related genes showed higher expression levels during the flowering and fruiting stages compared to the harvesting stage (S6 Fig). In fact, these annotated genes are primarily associated with precursor substances for synthesizing the aforementioned metabolites, rather than coding genes related to structural modifications of stilbenes and phenanthrene compounds and their downstream structures. Therefore, further exploration is needed to understand the accumulation mechanism of stilbenes and phenanthrene compounds during the harvest period.

Transcription factors play a crucial role in the regulation of gene expression, and 14 MYB, 1 bZIP, and 4 WRYK transcription factors were identified from the transcriptome data (S3 Table). Furthermore, the correlation analysis results revealed a strong association (|r| > 0.8, *p* < 0.05) between these transcription factors and 19 differential metabolites as well as enzymes that encode the biosynthesis pathway of BSP (Fig 6B). Seven MYBs [MYB (Cluster-15341.38703), MYB (Cluster-15341.10799), MYB (Cluster-15341.7163), MYB (Cluster-15341.26570), MYB (Cluster-15341.23331), MYB (Cluster-15341.109108), MYB (Cluster-15341.106806)] and two WRKYs [WRKY (Cluster-15341.100255), WRKY (Cluster-15341.100813)] transcription factors exhibited a positive correlation with the genes involved in BSP biosynthesis pathways, such as GMPP (Cluster-15341.45187), HK (Cluster-15341.16659), SUS (Cluster-15341.43292), GPI (Cluster-15341.37010), pgm (Cluster-15341.35370). The expression of four MYBs [MYB (Cluster-15341.7373), MYB (Cluster-15341.10799), MYB (Cluster-15341.7163), and MYB (Cluster-15341.26570)] and two WRKYs [WRKY (Cluster-15341.100255), WRKY (Cluster-15341.100813)] showed a negative correlation with GMPP (Cluster-15341.45187).

Additionally, MYB (Cluster-27863.0) was also negatively correlated with HK (Cluster-15341.16659). MYB (Cluster-15341.7373) and WRKY (Cluster-15341.100268) were positively correlated with the accumulation of dactylorhin A, gymnoside III, gymnoside X, gymnoside IX, militarine. In addition, the compound 4-methoxyphenanthren-2,7-diol was positively correlated with three MYBs [MYB (Cluster-15341.23331), MYB (Cluster-15341.109108) and MYB (Cluster-15341.106806)]. MYB (Cluster-15341.7373) and WRKY (Cluster-15341.100268) were negatively correlated with the content of isorhapontigenin and gymnoside VIII, MYB (Cluster-15341.38703) was negatively correlated with the accumulation of Gigantol, 4-methoxyphenanthrene-2, 7-diol. And three MYBs (Cluster-15341.23331, Cluster-15341.109108 and Cluster-15341.106806) transcription factors were negatively correlated with the accumulation of batatasin III. The compounds gymnoside III, gymnoside X and gymnoside IX were negatively correlated with MYB (Cluster-15341.38703) (S4 Table).

## Discussion

The exploration of the relationship between metabolite accumulation and gene expression differences is of great significance in revealing the mechanism of quality formation in *B*. *striata*. Chen et al. [39] conducted a study on the transcriptome and metabolome of *B*. *striata* varieties with different yield variations, finding significant differences in gene expression and metabolite accumulation. Further investigation into the correlation between differential genes, metabolites, and yield phenotype is beneficial for cultivating high-quality, high-yield *B*. *striata*. Previous studies have shown that rhizosphere microorganisms play an important role in the accumulation of metabolites such as polysaccharides and militarine in *B*. *striata* [40], and Zou et al. [41] confirmed that the fungus *Aspergillus flavus* (1-G4) isolated from *B*. *striata* can significantly promote plant growth and development by up-regulating plant growth hormones in *B*. *striata*. Obviously, a variety of internal and external factors have influence on gene expression and metabolite accumulation in *B*. *striata*. Therefore, this study aims to explore the relationship between metabolite accumulation and related gene expression in *B*. *striata* from the perspective of transcriptome and metabolome at different growth stages.

The bioactive substance BSP exhibits a diverse range of pharmacological activities and has gained extensive clinical application in the treatment of hemorrhage and wound healing. In addition, it also has the characteristics of biocompatibility and biodegradability, making it a good medicinal excipient raw material. Animal studies have shown that BSP has the dual characteristics of "drug and excipient" [42], showing great potential for application and development in the pharmaceutical field. Tubers are known to be the traditional medicinal part of *B*. *striata*, which is rich in BSP. However, as *B*. *striata* grows and develops, the level of BSP in the tuber changes [43]. The polysaccharide content of *B*. *striata* tubers was quantified at three distinct growth stages in this study. The findings indicated that the BSP content reached its highest level during the fruiting stage in late September, but experienced a decline during the subsequent harvest stage in October. The BSP content at the harvest stage remained significantly higher than that at the flowering stage, despite an initial notable increase followed by a subsequent decrease (Fig 1A), consistent with previous research findings [44]. The literature suggests that the flowering period of *B*. *striata* occurs in April, while the fruiting period spans from May to September. The aboveground part gradually withers in October, while new tubers buds emerge [45]. The underground tubers gradually grow from the flowering stage to the fruiting stage, accompanied by the accumulation of polysaccharides [46], while the aboveground parts gradually wither from the fruiting stage to the harvesting stage, weakening the function of photosynthesis to accumulate organic components. However, new buds rapidly grow and differentiate, consuming tuber nutrients, resulting in a decrease in polysaccharide content [44]. Our results are consistent with the above reports. Therefore, from the perspective of polysaccharide content, perhaps the quality of harvested *B*. *striata* during the fruiting period is better.

The results of non-targeted metabolome of *B*. *striata* tuber showed significant differences in metabolites at three different developmental stages. In the first principal component, flowering stage was significantly different from fruit stage and harvest stage, while the difference in the number of metabolites between the fruit and harvest periods is significantly reduced (Fig 2). There were 154 and 159 differential metabolites in the GQ vs HQ comparison group and the CSQ vs HQ comparison group, respectively, and the number was relatively close, while there were only 86 differential metabolites in the CSQ vs GQ comparison group, which was half of the number of differential metabolites in the first two comparison groups. The differences in metabolites mentioned above may be related to the longer time intervals between the flowering period and the fruit and harvest periods (5 and 6 months, respectively), while the shorter sampling time intervals between the fruit and harvest periods (one month). In addition to the temporal differences in metabolite accumulation, the changes in environmental factors such as temperature, humidity, and light from flowering (April) to fruiting (September) are more influential than those experienced from fruiting to harvesting (October).

It was reported that polysaccharide, 2-isobutyl malate gluconoxy-benzyl esters and stilbenes were the main active chemical components of *Bletilla* genus [9]. Among them, stilbenes are a class of organic phenolic compounds, which are generally synthesized by phenylalanine through phenylpropane pathway [36]. This kind of compound is a special secondary metabolic product of plants, which can improve the ability of plants to resist external stress and is a natural plant antitoxin [47]. Unfortunately, the key enzyme of stilbene synthase (STS) was not annotated in the transcriptome data, but its precursor biosynthesis-related genes were highly expressed during the flowering and fruiting stages. Therefore, the relationship between the elevation of these precursor genes at flowering and fruiting stages and the accumulation of stilbenes compounds at harvest stage and the potential molecular mechanism need to be further studied. The biological efficacy of militarine has been extensively demonstrated in numerous studies, and it is abundantly present in *B*. *striata*. In 2020, Chinese Pharmacopoeia specified militarine as the index component for the content determination of *B*. *striata*. At present, some studies are still using militarine as reference to establish the fingerprint of *B*. *striata* or to determine the content of its components. In addition to the compound militarine, an increasing number of studies recommend the use of multiple indicator components to evaluate the quality of *B*. *striata* [48], such as Zhang et al. [48] evaluated the quality of *B*. *striata* harvested in different periods and locations by measuring the content of militarine, dactylorhin A, bletilloside A, and batatasin III. Our metabolomics data indicated that the content of militarine, gyroside III, and dactylohinA first increased and then slightly decreased, while the content of coelonin and blestriarene A gradually increased (Fig 3). The above results suggest that we may need to determine the harvesting period reasonably based on the differences in the corresponding active ingredients required for the clinical treatment of diseases by *B*. *striata*, combined with the cumulative changes in *B*. *striata* compounds.

Research has shown that the pathway of polysaccharides in *B*. *striata* is basically consistent with the biosynthesis pathway of polysaccharides in other plants, which is mainly divided into three steps: sucrose is converted into Uridine diphosphate Glucose (UDP-Glc), Guanosine diphosphate Mannose (GDP-Man) or Guanosine diphosphate Fructose (GDP-Fuc) under the action of related enzymes, and then these sugars are generated into NDP sugars under the action of related enzymes, and finally added to various sugar residue chains under the action of GTs to form polysaccharide complex units [49]. Niu et al. [50] explored the biosynthetic pathway of BSP polysaccharide by de novo sequencing the *B*. *striata* transcriptomes, and found 10 enzymes are involved in the biosynthesis of *B*. *striata* polysaccharide. It is speculated that the metabolic processes of UDP-Glc and GDP-Man are the key metabolic pathways of BSP. Our current work also found genes encoding 9 enzymes related to plant polysaccharide biosynthesis, and most of the enzymes had multiple coding genes (Fig 5A). According to literatures, BSP is mainly composed of β-1, 4-mannose and β-1, 4-glucose, with the ratio of 3.5:1 [51]. The mannose proportion of the two monosaccharides composed of BSP is higher than that of glucose. Therefore, the biosynthesis of *B*. *striata* polysaccharide requires more mannose molecules. The transcriptome results showed that most of the enzyme genes promoting UDP-Glc biosynthesis were highly expressed during the fruit and harvest stages, while the expression of GDP-Man synthase was higher during the flowering stage. However, the determination of polysaccharide content found a significant increase from flowering to fruit period, while a decreasing trend was observed from fruit to harvest period. The findings suggest that the biosynthesis of GDP-Man may play a pivotal role in BSP biosynthesis, and the heightened expression of genes associated with GDP-Man biosynthesis during the flowering stage could potentially enhance polysaccharide accumulation in *B*. *striata* (Fig 5B).

The spatiotemporal specific expression of genes is often regulated by transcription factors, thus we analyzed the transcription factors at different stages. MYB (v-myb avian mye1oblastosis viral oncogene homolog) is one of the most abundant and functionally diverse transcription factor families in plants. The MYB domain is usually composed of 1 to 4 adjacent incomplete repeat sequences (repeat, R), which can be divided into four subclasses according to the number of R in the domain. R2R3MYB, which contains two Rs, is a key factor in regulating plant growth and development, secondary metabolism and response to biotic and abiotic stresses [52]. It has been reported that MYB is involved in regulating the biosynthesis of plant polysaccharides [38, 53], He et al. [49] also identified MYB genes biosynthesized with water-soluble polysaccharide (WSP) in Phalaenopsis equestris and *Dendrobium officinale*. MRKY family and bZIP family are also involved in the regulation of plant secondary metabolism. In the transcription data, MYB family transcription factors, bZIP and WRKY family transcription factors were all detected, suggesting that these genes may play an important roles in the regulation of BSP (Fig 6). A total of 9 transcription factors (7 MYB and 2WRKY) positively regulate 5 key enzymes (GMPP, HK, SUS, GPI, pgm) in BSP pathway. 7 transcription factors (5 MYB and 2 WRKYs) were negatively correlated with 2 key enzymes in the BSP pathway (GMPP, HK). 5 transcription factors were positively correlated with the accumulation of 2-isobutyl malate gluconoxy-benzyl ester compounds, and 5 transcription factors were negatively correlated with the accumulation of stilbenes. However, the exact interaction between them needs further research and confirmation.

## Conclusion

In summary, we analyzed the changes in the metabolome, transcriptome, and polysaccharide content of *B*. *striata* tubers at different growth stages, in order to explore the biosynthesis pathway and regulatory network of BSP, as well as the relationship between secondary metabolites and medicinal quality. The results showed that the accumulation of polysaccharides and most 2-isobutyl malate gluconoxy-benzyl ester compounds in *B*. *striata* was higher during the fruit period, while stilbenes such as coelonin, 3’-O-methylbatatasin III and blestriarene A were higher during the harvest period. The transcriptome results showed that the expression pattern of GDP-Man biosynthesis related enzyme genes correspond to the cumulative changes in BSP, indicating that the biosynthesis of GDP-Man plays an important role in promoting BSP biosynthesis. Meanwhile, it was found that multiple transcription factors were positively correlated with polysaccharide biosynthesis related genes and the accumulation of 2-isobutyl malate gluconoxy-benzyl ester compounds, implying that these transcription factors have a regulatory effect on the quality formation of *B*. *striata*. We will focus on conducting in-depth and systematic research on these interactions in our future work, in order to provide scientific basis for the cultivation of high-quality traditional Chinese medicine *B*. *striata*.

## Supporting information

S1 Fig Total ions current in *B*. *striata*. (TIF)

S2 Fig OPLS-DA scores and volcano maps among three different comparison groups. (TIF)

S3 Fig The unigene length distribution map and BUSCO assessment results. (TIF)

S4 Fig GO annotation classification statistics and KOG annotation classification statistics. (TIF)

S5 Fig Classification of transcription factor families. (TIF)

S6 Fig Expression analysis of genes involved in the phenylpropanoid biosynthetic pathway. (TIF)

S1 Table Compounds of *B*. *striata* in three different stages. (XLSX)

S2 Table Summary of sequences analysis of 9 libraries. (XLSX)

S3 Table List of the genes associated with BSP biosynthesis and list of transcription factor. (XLSX)

S4 Table Correlation analysis of TFs, BSP-related genes and 19 differential metabolites. (XLSX)
