# Supplementary figures and images for: Combined metabolomics and transcriptomics reveal the secondary metabolite networks in different growth stages of Bletilla striata (Thunb.) Reichb.f
Source: PLoS One. 2024 Jul 24;19(7):e0307260. doi: 10.1371/journal.pone.0307260 (PMC11290943; doi:10.1371/journal.pone.0307260)

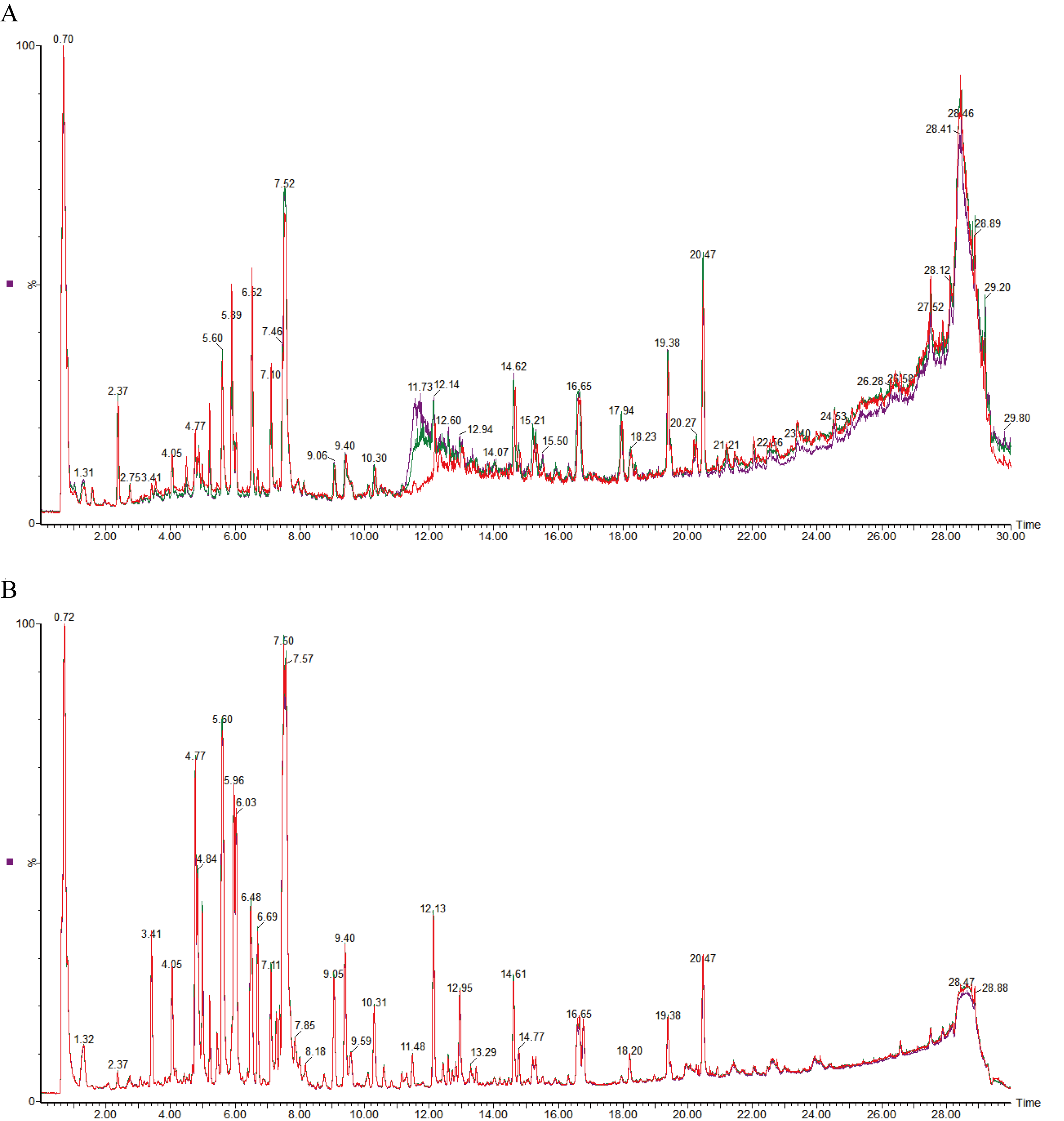

Supplement: S1 Fig — (TIF) [file pone.0307260.s001.tif]

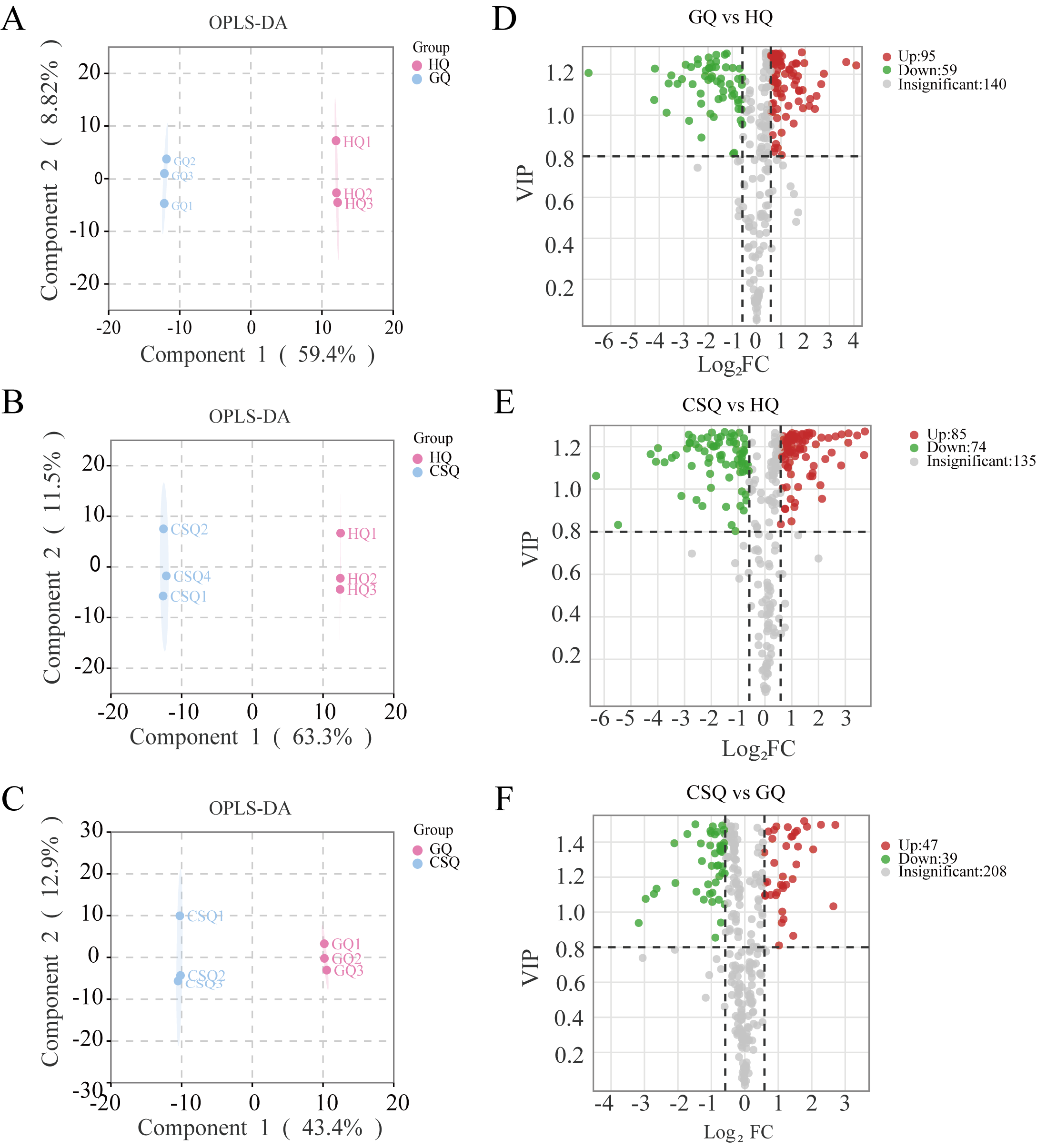

Supplement: S2 Fig — (TIF) [file pone.0307260.s002.tif]

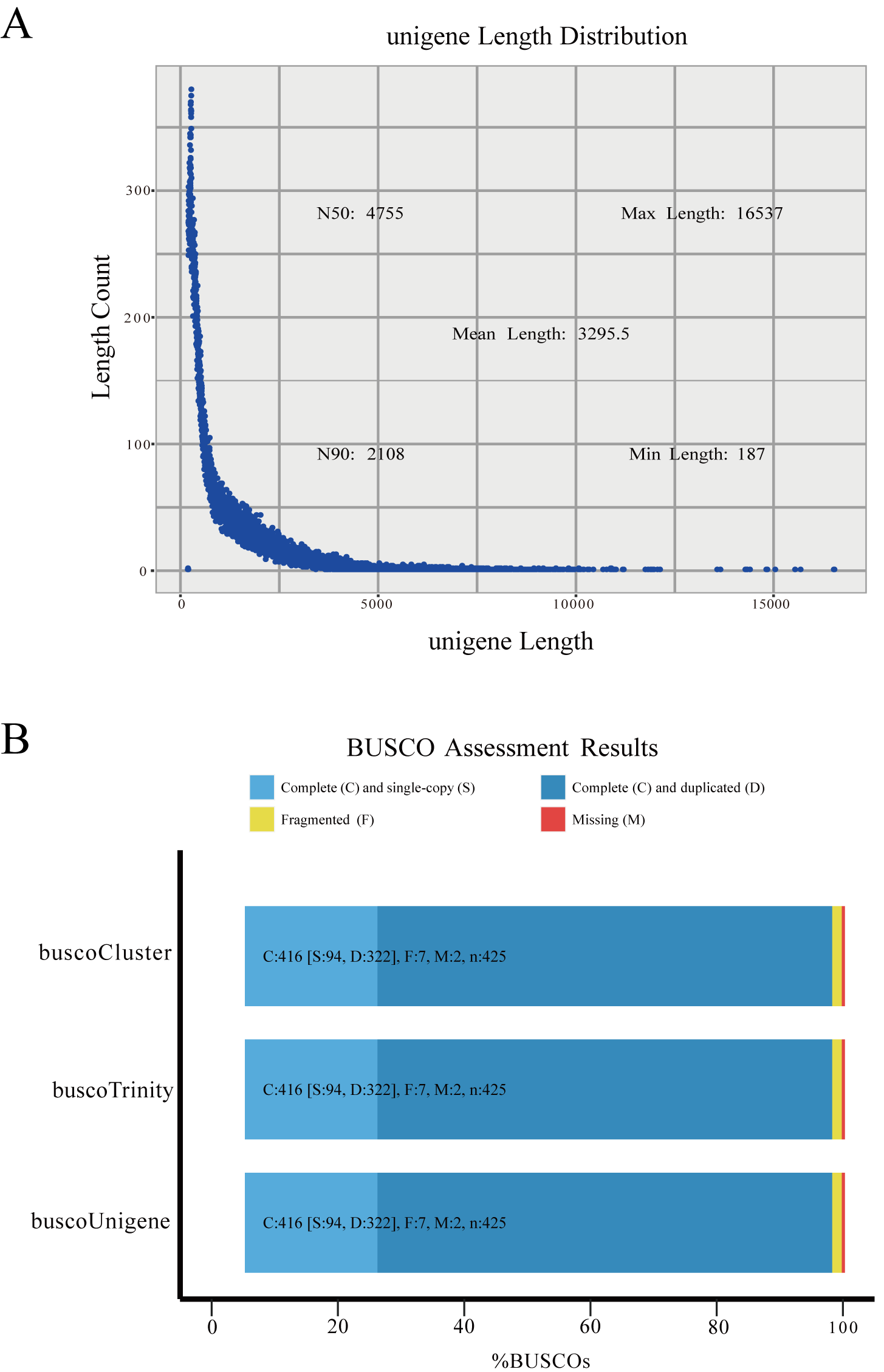

Supplement: S3 Fig — (TIF) [file pone.0307260.s003.tif]

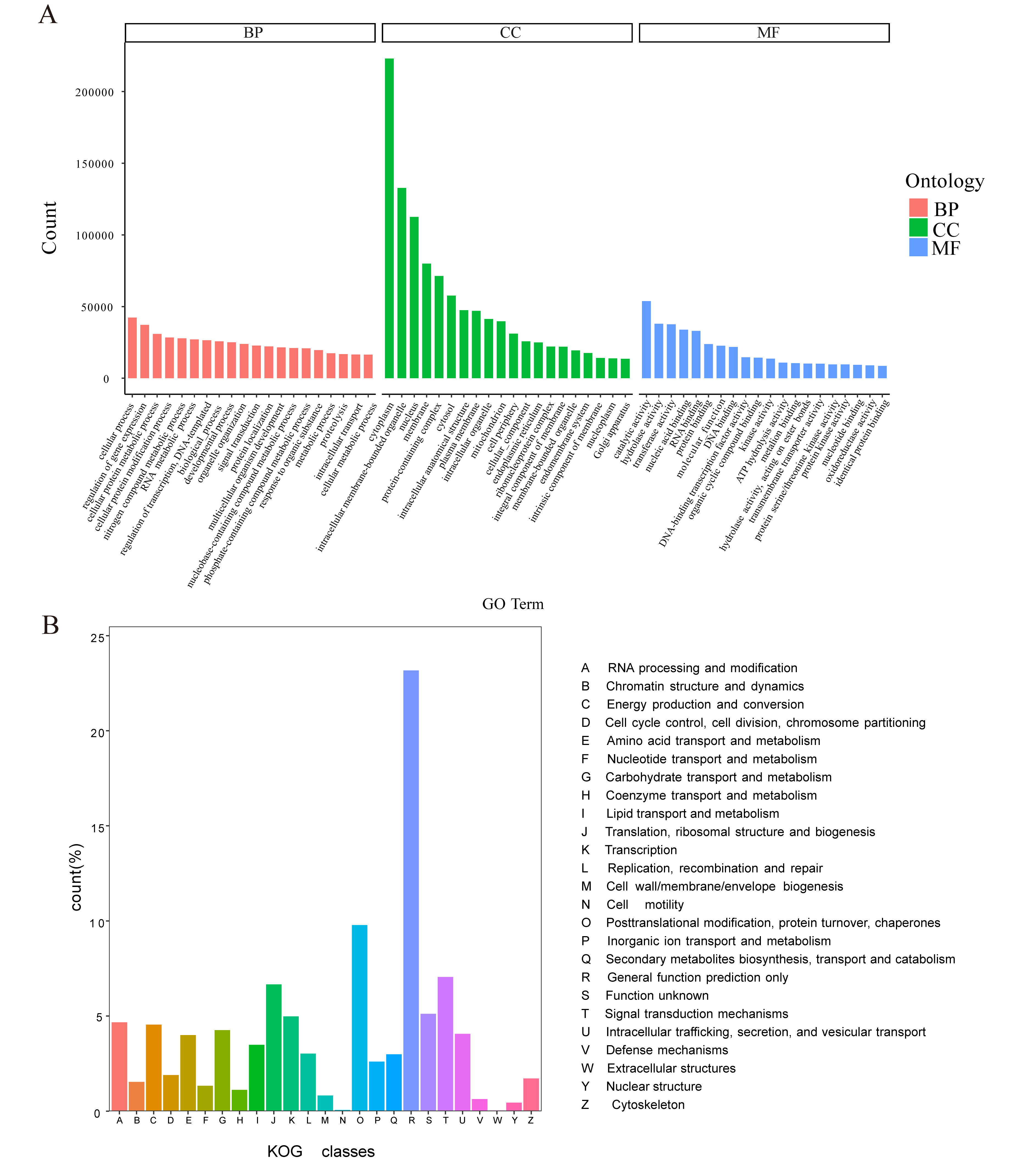

Supplement: S4 Fig — (TIF) [file pone.0307260.s004.tif]

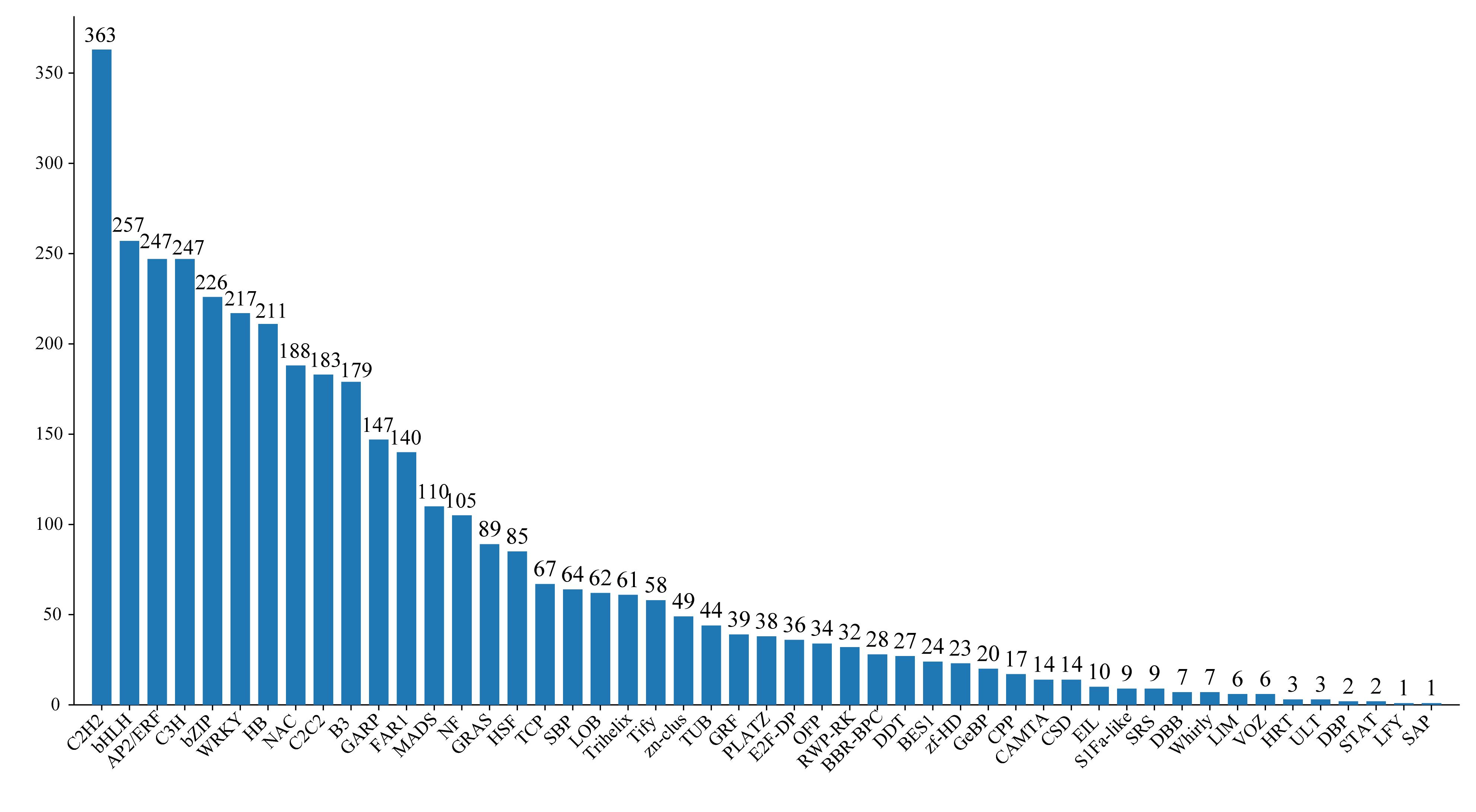

Supplement: S5 Fig — (TIF) [file pone.0307260.s005.tif]

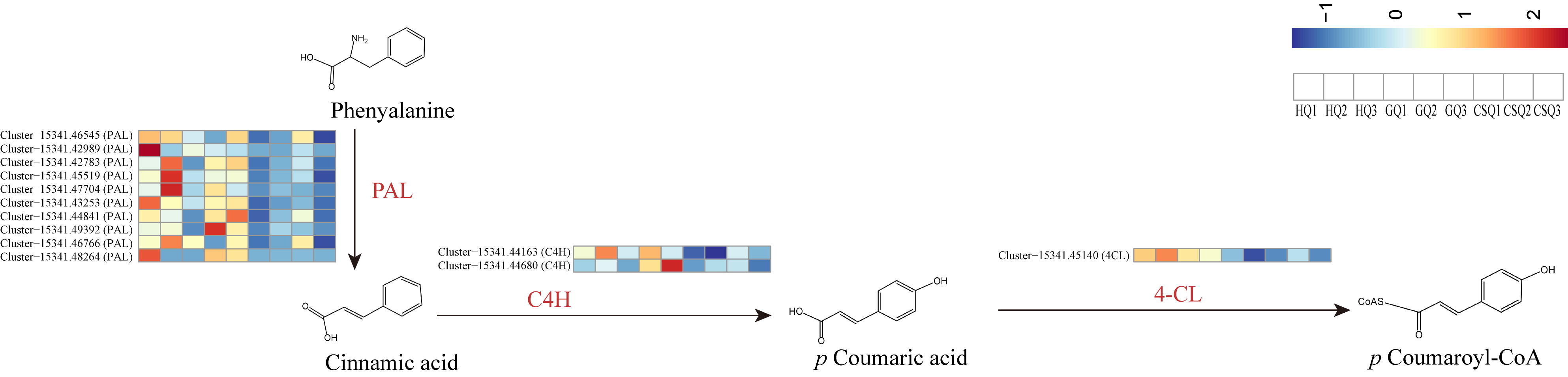

Supplement: S6 Fig — (TIF) [file pone.0307260.s006.tif]
